# Ailanthone: a new potential drug for castration-resistant prostate cancer

**DOI:** 10.1186/s40880-017-0194-7

**Published:** 2017-03-03

**Authors:** Shihong Peng, Zhengfang Yi, Mingyao Liu

**Affiliations:** 1 East China Normal University and Shanghai Fengxian District Central Hospital Joint Center for Translational Medicine, Shanghai Key Laboratory of Regulatory Biology, Institute of Biomedical Sciences and School of Life Sciences, East China Normal University, 500 Dongchuan Rd, Shanghai, 200241 P.R. China; 2 Department of Molecular and Cellular Medicine, Center for Cancer and Stem Cell Biology, Institute of Biosciences and Technology, Texas A&M University Health Sciences Center, Houston, TX 77030 USA

Prostate cancer (PCa) is the most common male cancer [1, 2]. PCa initially depends on androgen receptor (AR) signaling for growth and survival. Androgen deprivation therapy causes a temporary reduction in PCa tumor burden, but the tumor eventually develops into castration-resistant prostate cancer (CRPC) with the ability to grow again in the absence of androgens [3]. Mechanisms of CRPC progression include AR amplification and overexpression [4], *AR* gene rearrangement promoting synthesis of constitutively-active truncated AR splice variants (AR-Vs) [4], and induction of intracrine androgen metabolic enzymes [3]. Current anti-androgen therapies including MDV3100 (Enzalutamide) and abiraterone have focused on the androgen-dependent activation of AR through its ligand-binding domain (LBD), but do not provide a continuing clinical benefit for patients with CRPC and presumably fail due to multiple mechanisms including the expression of AR-Vs lacking the LBD [5]. These AR-Vs signal in the absence of ligand and are therefore resistant to LBD-targeting AR antagonists or agents that repress androgen biosynthesis [6].

To identify compounds that block the transcriptional activities of both ligand-dependent full-length AR (AR-FL) and AR-Vs, we used the MMTV-luciferase (MMTV-luc) reporter system that contains AR-binding elements [7] to screen approximately 100 compounds from a library of natural compounds from Traditional Chinese Medicine and identified a small-molecule compound termed ailanthone, which is extracted from the whole seedlings of *Ailanthus altissima* (Simaroubaceae) that has antimalarial and antitumor activities [8]. In our work recently reported in the paper entitled “Ailanthone targets p23 to overcome MDV3100 resistance in castration-resistant prostate cancer” in *Nature Communications* [9], ailanthone not only blocked the ligand-induced activities of full-length AR but also inhibited AR-V which lacks the LBD at low concentrations (AR-FL half maximal inhibitory concentration [IC_50_] = 69 nmol/L, 95% confidence interval [CI] = 53–89 nmol/L; AR_1–651_ [a constructed AR splice variant] IC_50_ = 309 nmol/L, 95% CI = 236–687 nmol/L in 22RV1 cells). Ailanthone decreased the androgen-dependent induction of endogenous AR downstream genes *PSA*, *TMPRSS2*, *FKBP5*, *SLC45A3*, and *NDRG1* mRNA expression in LNCaP cells. To investigate whether ailanthone suppressed the functioning of AR-V which lacks the LBD, we performed RNA sequencing after treating LNCaP cells with or without ailanthone in the absence or presence of AR_1–651_. Indeed, ailanthone strongly suppressed AR_1–651_-induced expression of some genes, such as *PSA* and *TMPRSS2*, supporting the potential therapeutic use of ailanthone in CRPC. Moreover, ailanthone reduced the expression of both the full-length AR and the AR-Vs in vitro and in vivo. Using the sulforhodamine B colorimetric (SRB) assay, we confirmed that ailanthone potently inhibited the growth of several PCa cell lines including LNCaP, c4–2b, 22RV1, and LAPC4. Interestingly, ailanthone more potently inhibited the growth of AR-positive PCa cells than either AR-negative tumor cell lines or normal prostate cell lines. Notably, not only intraperitoneal injection administration but also per os administration of ailanthone had excellent efficiency for blocking the growth and metastasis of CRPC in LNCaP, 22RV1, and VCaP subcutaneous xenografts and 22RV1-luc orthotopic xenografts.

Our study also explored the mechanism of ailanthone-induced AR degradation [9]. We found that ailanthone disrupted the interaction between AR and the chaperones HSP90, HSP70, and HSP40, and consequently AR was ubiquitinated and degradated through the proteasome-mediated pathway. When not bound to ligand, AR resides in the cytosol bound to the foldosome, a complex of heat shock, chaperone, and co-chaperone proteins including HSP90, HSP70, HSP40, and p23. Ailanthone could bind to p23 protein which is very important for the stabilization of the HSP90-client complex, and ailanthone prevented the interaction of HSP90 with p23. Given that ailanthone was able to bind to p23 and knockdown of p23 substantially prevented ailanthone-induced cell growth arrest, we propose that ailanthone induces AR degradation through binding to p23 and disrupting the HSP90-client complex. Indeed, PCa cells that express AR showed greater sensitivity to ailanthone at lower concentration, suggesting the degradation of AR by ailanthone plays a major role in inhibiting the growth of AR-positive PCa cells at low concentrations of ailanthone. Therefore, we conclude that targeting p23 is the major mechanism of ailanthone and that ailanthone-induced AR degradation is at least a critical mechanism of ailanthone-dependent cell growth inhibition in PCa. However, how ailanthone regulates the molecular conformation of p23 and prevents the interaction of p23 with HSP90 remains undetermined in our work. Clarifying the mechanism of ailanthone remains to be further investigated.

In pharmacokinetic studies, ailanthone exhibited good solubility in water and good bioavailability (>20%). We addressed some key safety issues of ailanthone, such as cytochromes P450 (CYPs) inhibition and hepatotoxicity. The current study showed that ailanthone had no obvious inhibitory effects on the main CYPs in humans and rats. In addition, ailanthone did not influence the expression of CYP enzymes and had no significant hepatotoxicity after a 5-day administration in the present study. Therefore, ailanthone would have a low potential to cause possible toxicity and drug–drug interactions in which CYP enzymes are involved, suggesting a sufficient safety window for its putative use as a promising anticancer agent. Meanwhile, various physicochemical properties of ailanthone calculated on the ACD/I-Lab showed that the physiochemical parameters of the natural compound ailanthone met with “Lipinski’s Rule of Five” [10]. Our results suggest that ailanthone can be developed as a potential drug candidate with various drug formulations because of its ideal solubility and bioavailability.

In conclusion, we screened and characterized ailanthone, a novel compound with excellent drug-like characteristics that is able to overcome MDV3100-resistance in PCa cell lines. Ailanthone was efficacious in suppressing the growth and metastasis of CRPC via targeting p23. As a result, ailanthone can be considered a new potential drug candidate for PCa, and it is worthy of further research and investigation.
